# The Cell as the First Niche Construction

**DOI:** 10.3390/biology5020019

**Published:** 2016-04-28

**Authors:** John S. Torday

**Affiliations:** Evolutionary Medicine, UCLA, Westwood, CA 90502, USA; jtorday@ucla.edu; Tel.: +1-310-222-8186; Fax: +1-310-222-3887

**Keywords:** niche construction, evolution, teleology, epigenetic

## Abstract

Niche construction nominally describes how organisms can form their own environments, increasing their capacity to adapt to their surroundings. It is hypothesized that the formation of the first cell as ‘internal’ Niche Construction was the foundation for life, and that subsequent niche constructions were iterative exaptations of that event. The first instantation of niche construction has been faithfully adhered to by returning to the unicellular state, suggesting that the life cycle is zygote to zygote, not adult to adult as is commonly held. The consequent interactions between niche construction and epigenetic inheritance provide a highly robust, interactive, mechanistic way of thinking about evolution being determined by initial conditions rather than merely by chance mutation and selection. This novel perspective offers an opportunity to reappraise the processes involved in evolution mechanistically, allowing for scientifically testable hypotheses rather than relying on metaphors, dogma, teleology and tautology.

## 1. Introduction

The following is a contribution to Biology (Basel)’s “Beyond the modern synthesis—What have we missed?” As such it addresses how emerging concepts in evolution theory—Niche Construction Theory, Social Networking and Stigmergy—all complement and facilitate a novel Central Theory of Biology [1].

Niche construction describes how macro organisms form their own environments [2], increasing their capacity to adapt to their surroundings. However, perhaps that process is actually an exaptation [3] of how cells evolved in the first place by forming their own internal niche construction through endosymbiosis [4]. If that is the case, this is one of the first conceptual ways in which evolution and ecology have formally been merged to great fanfare as these disciplines have largely remained isolated from one another [5]. Now, with the re-emergence of Lamarckian epigenetics [6], Niche Construction Theory is ever more relevant since these two mechanisms naturally complement, reinforce and synergize one another [7].

The formation of micelles from the lipids contained within the asteroids that struck the nascent Earth to form the oceans [8] may have provided the first niche in which chemiosmosis [9] provided the internal environment for negentropy [10], circumventing the Second Law of Thermodynamics, perpetuating life under the auspices of homeostasis [11]. Thus, as unicellular organisms dispersed across the Earth they fashioned their own external environments, ultimately generating the Earth’s atmosphere. It was the atmospheric changes—increased oxygen [12] and carbon dioxide [13]—that caused the selection pressure for metabolic drive [13], generating metazoans beginning about 500 million years ago. The Greenhouse Effect caused by rising levels of atmospheric carbon dioxide produced by cyanobacteria caused the drying up of ponds, lakes, rivers and oceans, forcing water dwelling vertebrates on to land [14], or to become extinct. The subsequent undulations in oxygen in the atmosphere, ranging between 15% and 35% [15] caused physiologic stress, beginning with the leaking of calcium from the endoplasmic reticulum [16] in unicellular eukaryotes, hypothesized to have given rise to the peroxisome [17]. Subsequent stresses were exaptations of the homologous calcium-lipid epistatic balancing mechanism, forging ancestral and contemporary vertebrates, including hominids [18].

## 2. Compartmentalization of Physiologic Traits—Endosymbiosis

Key to understanding this internally consistent mechanism of evolution is the compartmentalization of physiologic traits, beginning with the protocell [19]. The internalization of the cell membrane initiated chemiosmosis [20,21], the partitioning of ions within the cell on either side of a semi-permeable membrane, causing ion flux as the origin of bioenergetics [22]. This allowed the protocell to reduce the entropy within it, or negentropy, enabling it to circumvent the Second Law of Thermodynamics, existing far from equilibrium, governed by homeostatic control of calcium/lipid balance [23]. The subsequent formation of internal organelles such as the nucleus, endoplasmic reticulum and Golgi Apparatus were all a consequence of endosymbiosis [24]. The subsequent evolution of eukaryotes was facilitated by the advent of cholesterol in the plasmalemma [25], promoting metabolism, respiration and locomotion, the mainstays of vertebrate evolution [26]. Thus, for example, when rising oxygen levels in the atmosphere caused endoplasmic reticulum stress, the evolutionary ‘reply’ was the formation of the Peroxisome [17].

Hence, this process constitutes the first Niche Construction, bearing in mind that the ‘niche’ is internal, not the traditional way in which niche construction is thought of as the organism fashioning its own external environment. However, bear in mind that evolution is characterized by serial exaptations [27]. The formation of the cell membrane delineated internal and external as the origin of life—its ability to self-organize is a function of this self-referential property, repeated iteratively [27]. This insight to the origin and causation in biology is critical to understanding the principles of evolution [7,11,18,25,27].

## 3. Niche Construction + Epigenetics = Evolution

At some point in evolution, nucleotides were exploited as the memory system of the cell. Experimentally, nucleotides were shown to be produced by passing and electric charge through the primitive Earth atmosphere [28], providing the putative source of DNA and RNA. In addition by applying endosymbiosis theory, these nucleotides were advantageous in reminding the organism which historic phenotypic traits it used under what conditions molecularly. Perhaps, however, even more importantly, the derivation of nucleotides from the environment is significant because it may have been the archetype for epigenetic inheritance, the acquisition of epigenetic marks from the environment being assimilated by the germline cells. In the context of niche construction, the ability of the organism to effectively engage the environment may be the actual mechanistic purpose for the phenotype, i.e. that it is not merely the chance result of the combination of the parental genetics, but the way in which the environment instructs the progeny [27]. 

A classic example is the discovery of an orchid with an exceptionally long nectary (30 cm) that was seemingly too deep for insects to pollinate, challenging Darwin’s theory that there must be such an organism [29]. Eventually, however, Darwin identified a sub-species of giant Congo moth from Madagascar with an amply long tongue, solidifying his selection theory of evolution. Conversely, this co-evolution of flower and moth may have been the result of the interaction between niche construction and epigenetics affecting both rather than random mutation and selection. Again, chance mutation is not scientifically testable, whereas niche construction interacting with epigenetics is.

## 4. Phenotypic Variation as Agency for Epigenetic Inheritance

**Scheme 1:**
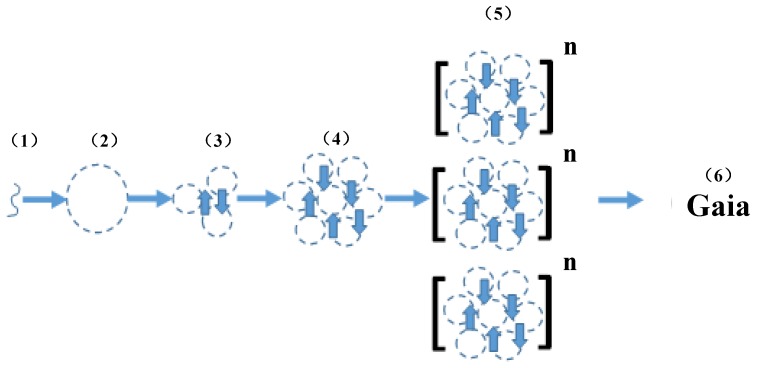
Interaction between Niche Construction and Epigenetic Inheritance. (**1**) lipids in water spontaneously form micelles (**2**) delineating the internal niche construction of the protocell; (**3**) communication between cells promotes cell-cell communication; (**4**) metabolic cooperativity gives rise to multicellular organisms; (**5**) positive interactions between niche construction and inheritance of epigenetic marks fosters the formation of organismal communities; (**6**) the widespread interactions between niche construction and epigenetics ultimately gives rise to Gaia.

The notion that niche construction fosters evolution by generating circumstances for epigenetic interactions (see Scheme 1) is an attractive alternative to the ‘chance’ nature of evolution. By determining phenotypic variation, epigenetics solidifies and expands the environmental niches in which the organism can live, at the same time constraining the opportunities for the acquisition of novel epigenetic marks. Seen as an active ‘agent’ for the acquisition of epigenetic marks, the phenotype takes on a very different role from the one depicted in Darwinian evolution. In the former case, phenotypic change over time has been in service to the effective integration of the organism with its environment in order to continuously monitor for external changes; in the case of the latter, random mutations change the adults, affecting their reproductive success. The net result of phenotypic agency for collecting epigenetic marks is what is classically described as the ‘emergence and contingence’ of evolution, but now with a mechanistic explanation for how and why that is the case. This sea change in the way we think of niche construction and epigenetic inheritance as an interactive process that has facilitated exaptations since the inception of life itself is a powerful way of understanding the predictive capacity of evolution. Seen from the cellular-molecular perspective, rather than as organisms reproducing optimally to transfer their genetics to the next generation, evolution can finally be seen as a comprehensively integrated process of adaptation without needing to resort to tautologic, teleologic, dogmatic metaphors like Natural Selection, Descent with Modification and Survival of the Fittest [30].

## 5. Niche Construction + Epigenetics= Primacy of the Unicellular State

Once the niche construction/epigenetic mechanistic basis for evolution is recognized, the life cycle can, for example, be seen mechanistically traversing from zygote to zygote instead of from adult to adult [31]. The key is in appreciating that the organism is perpetually trying to maintain its homeostatic equipoise in an ever-changing environment, monitoring its condition using epigenetics to affect the phenotype, which in turn is acting as a probe through niche construction. However, the organism is always ensuring that it does not deviate from the first principles of physiology by returning to the unicellular germline cells, undergoing meiosis and passing through embryogenesis to ensure homeostatic fidelity [32].

Given that reproduction is a means of processing epigenetic marks, that the life cycle is a means for determining epigenetic exposure to the environment, and that the phenotype is a means of effectively acquiring epigenetic marks [27], not merely as the ends they are seen as through the lens of descriptive biology renders evolution a useful, predictive tool rather than merely a “just so story” [30,33].

## 6. Gaia Theory = Niche Construction + Epigenetics

Gaia theory envisions the Earth as a self-regulating complex system involving the biosphere, the atmosphere, the hydrosphere, and the pedosphere, tightly coupled together in a perpetually evolving system [34]. As a holistic entity, Gaia seeks a physical and chemical environment optimal for contemporary life [35].

Gaia evolves through a cybernetic feedback system mediated by the biota, leading to stable conditions for habitability in full homeostasis. The Earth’s surface is essential for the optimal conditions of life, depending on interactions between life forms, especially microorganisms [36], with inorganic elements. These processes determine a global control system that regulates the Earth's surface temperature, atmospheric composition and ocean salinity, driven by the force of the global thermodynamic disequilibrium of the Earth system [37].

Planetary homeostasis fostered by living forms had previously been recognized in the field of biogeochemistry [38], and it is also being investigated in other fields such as Earth system science [39]. Gaia theory is novel because it relies on the precept that homeostatic balance is an active process with the goal of maintaining the optimal conditions for life [40].

Perhaps equally important from a humanistic standpoint is the potential for a new appreciation of our biologic origins and heritage. The mere fact that all biota are interrelated and are the products of their interactions with Mother Earth should provide a more ecumenical attitude towards both. James Lovelock called this attitude adjustment Gaia Hypothesis, but he was appealing to our collective conscience. However, a fundamental understanding of who we are as a species among species, forged by the physical environment is a much wanted game changer [41], given the continued belief in the Anthropic Principle [42]—that we are in this environment, not of it [18,25,27,41].

Gaia theory is complemented by more recent theories of how the biota are integrated with the earth into one unified whole. For example, Stigmergy [43] is a proposed mechanism for traces left in the environment by actions stimulating subsequent actions, providing a way to understand the existence of emergent coherent systematic activities such as social networks [44].

Stephen J. Gould famously asked whether if we replayed the evolution tape we would recapitulate it [45]. The answer, based on the idea that we have evolved from and in response to the ever-changing environment is a resounding NO. Sure, we have experienced a Greenhouse Effect in the past, complete with rising sea levels, but if we have to undergo that process again because of Global Warming our phenotype will have to change or we will probably become extinct. Furthermore, even populating another ‘Earth’ would not solve the problem if we do not face ourselves and recognize how and why we have come to this place and time in human history.

## 7. Niche Construction Controversy

Niche construction advocates argue that it is distinctly different from conventional Darwinian evolution theory [46,47]. For the latter, evolutionary processes are those that change gene frequencies, as is the case for natural selection, genetic drift, mutation and migration. Darwinists do not see how niche construction can either generate or filter genetic variants without the aid of these mechanisms for independently changing gene frequencies [46,47]. Conversely, niche constructionists take a more inclusive view of the evolutionary process that does not directly affect gene frequencies per se. The merging of niche construction and epigenetic inheritance is such a liberal perspective that would facilitate a fuller understanding of evolution. It lends itself to an understanding of evolution as a continuous process, integrating environmental and biologic properties through complementary properties of the organism.

It should be noted here that there is a fundamental difference between Darwinian evolution theory and the concept that the cell was the first niche construction. The former is predicated on random mutation and natural selection as the mechanism of evolution, whereas the latter is deterministic, contingent on the First Principles of Physiology that dictated the self-organization of the first cell [7,18,25,27].

Such an accommodating view of evolution would, for example, resolve the debate between gradualism and punctuated equilibrium—evolution is actually both. Like Niels Bohr’s resolution of light as wave or particle, he explained that it was both; it was just a matter of how light was measured [48].

## 8. Conclusions

Ernest Rutherford famously stated that “All science is either physics or stamp collecting”. The relevance of that statement is no more important in biology than in any other scientific discipline. Unfortunately, biologists do not recognize that describing a mechanism is not the same as knowing how it actually works [49]. Nowhere is that more apparent than in evolution theory, which is composed of metaphoric Just So Stories instead of testable, refutable, hypothesis testable science. However, knowing our origins and trajectory is critically important to understanding the human condition. In order to make this point, herein two relatively new ideas in biology have been merged together to demonstrate the difference between descriptive and predictive science. 

It was Thomas Kuhn [50], the author of “The Structure of Scientific Revolutions” who said that the hallmark of a paradigm shift in science is when the language changes—such redefinitions herald a sea change in biology. In that spirit, several standard terms in biology have previously been redefined, such as homeostasis [11], cell [25], pleiotropy [51], and heterochrony [52] to point out the value added in understanding the mechanistic basis for biology.
